# Essentials of Physics and Chemistry

**Published:** 1889-05

**Authors:** 


**Essentials of Physics and Chemistry.** Written especially for the use of students in medicine. By CONDICT W. CUTLER, M. S., M. D., Physician-in-Chief of the New York Dispensary; Instructor in Diseases of the Skin at the New York Post-Graduate School and Hospital, etc. Third edition, enlarged and revised. Cloth, 12mo, pp. ix., 296. New York and London: G. P. Putnam's Sons. 1889.

While the further multiplication of compends is not to be encouraged, inasmuch as they certainly tend to make superficial students, still one must confess that in the case of Dr. Cutler's book more than the average grade of excellence in books of its kind has been attained. Its chief fault is the one commonly noted—when an author attempts to treat a vast subject in a small compass—viz.: condensation at the expense of clearness. This is the most marked in the case of definitions. Again, we cannot understand why a subject so important as animal chemistry should have been given so small a space—only three pages. Albuminous principles, for instance, receive barely twenty full lines, and the other parts of this branch of chemistry are proportionately abbreviated. The chemical portion of the work, however, is on the whole better done than the physical. Typographically the book is excellent, and its convenient size will make it popular with medical students, who no doubt will find it just what the author intended it to be—an aid in preparing for "quiz" or examination.

